# Construction and validation of a short-form Quality-Of-Life Scale for Chinese Patients with Benign Prostatic Hyperplasia

**DOI:** 10.1186/1477-7525-7-24

**Published:** 2009-03-17

**Authors:** Yanfang Guo, Jingcheng Shi, Ming Hu, Zhenqiu Sun

**Affiliations:** 1 Department of Epidemiology and Health Statistics, School of Public Health, Central South University, Changsha, Hunan, PR China; 2 Huaihua Medical School, Huaihua, Hunan, PR China

## Abstract

**Background:**

In 2003, a 74-item quality-of-life (QOL) scale for Chinese benign prostatic hyperplasia (BPH) patients (BPH-QLS) was developed. Although the scale displayed good reliability and validity, the time required to complete it may limit its use. The purpose of this study was to construct and validate a short-form quality-of-life (QOL) scale for Chinese patients with Benign Prostatic Hyperplasia (BPH).

**Methods:**

According to the previously published 74-item quality of life scale for BPH (BPH-QLS), we developed a pool of items, then condensed these items and validated the shortened scale, based on collected data from 163 patients with BPH. We used eight methods to reduce the items.

**Results:**

A 33-item QOL scale for BPH (short-form of BPH-QLS) was constructed. The time take by the new scale was much shorter than the original one. There was no significant difference between the 33-item scale and 74-item scale, in terms of reliability. Moreover, the 33-item BPH-QLS showed a high correlation with the 74-item BPH-QLS (r = 0.971). Scores generated by the two scales were not only parallel and coincident but also at the same level.

**Conclusion:**

We concluded that the reliability and validity of the short form of BPH-QLS is close to those of the 74-item BPH-QLS. It should be a good choice in clinical practice for its greater compliance and clinical feasibility.

## Background

BPH is a common male disorder that, though rarely life-threatening, greatly affects patients' perceived quality of life (QOL). QOL is an important component in the evaluation of BPH treatment strategies. Several BPH-specific QOL scales have been developed, e.g., the International Prostate Symptom Score (IPSS), the Danish Prostate Symptom Score (DAN-PSS-1), the International Continence Society 'male' questionnaire short-form (ICSmale-SF) and the BPH Quality of Life Index [1-4]. Although the IPSS and QOL index are universally used, they can only quantify severity of lower urinary tract symptoms suggestive of BPH and evaluate treatment efficacy, and can not fully reflect the overall quality of life. Moreover, because QOL scale depends on the culture background, it is necessary to develop a Chinese version of the scale. In 2003, a 74-item BPH-QLS with five domains (disease, physical, social, psychological, satisfaction) was developed for Chinese BPH patients. Although the scale displayed good reliability and validity [5,6], the time required to complete it may limit its use. Demands for efficiency, reduced respondent burden, greater compliance, and clinical feasibility have led to the development of shorter questionnaires. The aim of this study was to construct and validate the short-form of BPH-QLS and to compare its results with those of the original instrument.

## Patients and methods

This study was conducted in the city or provincial hospital in Changsha, the capital city of the Chinese Province of Hunan, from March 2005 to December 2005. We stratified the recruitment by patient sources, so that a balance number of study subjects can be obtained from inpatient, outpatient, and community based patient settings. Male patients who came to the participating hospitals for treatment or physical examination for lower urinary tract symptoms (LUTS) suggestive BPH were approached by research assistants to participate into the study. All the participating patients were assessed by the research assistants and only those patients who were considered competent were recruited. The research assistants explained to the patients of the purpose of the study and signed consents were obtained from the patients if they agreed to participate into the study after full explanation. Attentive digital rectal exam (ADRE) or transrectal ultrasound (TRUS) was used to assess benign prostatic enlargement. Using the recommendations of the Fourth International Consultation on BPH, patients with the following conditions were excluded: aged< 50 years; with prostate cancer; previously failed invasive treatment for BPH; possibility of a neurological disease; surgical or wound history related to the pelvic cavity; history of venereal disease; taking drugs affecting bladder outlet function; or unable to adequately express feelings. Self-filled questionnaires for the full BPH-QLS, SF-36, IPSS were distributed to the study subjects. In addition, a control group with no past or current history of BPH randomly selected among the community care settings matched by age and educational level were asked to complete the questionnaires. This study was approved by the Ethics Committee of Central South University.

### Scale construction

#### Phase 1

After consulting relevant published QOL scales for BPH, such as IPSS, DAN-PSS-1, ICS-BPH, BII, SPI, BPHQOL, WHOQOL-100, BPH Quality of Life Index, BPH-HRQOL and BSP-BPH [1-4,7-11], we added 12 new items to the 74-item BPH-QLS according to the consensus reached by 18 relevant experts. An initial draft-item pool (86 items) was then generated (see additional file 1).

#### Phase 2

Initial expert score was made by a panel consisting of 18 members (specialists in urology, nursing, psychology, statistics, and public health and BPH patient representatives) according to the importance on a scale of 1–5 (1 = least important and 5 = most important). Final score was determined by 12 of the panel members after further review and consultation.

#### Phase 3

Eight (8) statistical methods of analysis were used to select items in data collected form 163 BPH patients. The first method was the scoring items by experts. Items which had total score < 40 (full score was 60 for 12 experts finished the consultation at the end) were deleted [12]. The second method used coefficient of variation. Items with a variation coefficient < 25% for each domain were deleted [12]. The third method used of discriminatory analysis to retain items which could distinguish between men with BPH and men without BPH. The fourth method involved the use of correlation coefficient to eliminate unimportant items in each domain [2]. High item-to-item correlation was defined by a coefficient > 0.8. The fifth method involved the use of multiple stepwise regression to eliminate non-significant items in each domain (*α*_entry _= 0.10, *α*_exit _= 0.15) [9]. The sixth method used Cronbach'α coefficient. The last 50% of items which induced an increase of Cronbach'α coefficient in each domain were deleted [13]. The seventh method involved the use of factor analysis to delete the last 50% of items with low factor loading [13]. The eighth method involved the use of cluster analysis to retain representative items.

#### Phase 4

Items which selected by at least 6 of the 8 analysis methods were retained in the scale, while the items selected by only 5 of the 8 methods were retained only if recommended by specialist.

### Scale scoring

The short form of BPH-QLS with 5 points and of equal interval (1 low 5 high) was used for the scoring of items, which included 32 reverse items. Patients were then asked to select the relevant point on the scale based on their perceptions. Primitive scores were subtracted from six to get a new score. After the score was renewed, the higher score indicated the better quality of patient's life.

### Scale validation

The major reliability and validity tests were used to validate the short new QOL instruments [6]. Correlation coefficients (CCs) were calculated for the original and 1-week repeat scale and each domain for test-retest reliability. Internal consistencies for the instrument and its domains were assessed by Cronbach'α coefficient. The validity of the short form of BPH-QLS was tested in three aspects. For structure validity, we used exploratory factor analysis and correlation analysis. For criterion validity, two criteria (SF-36 and IPSS) were used. For discrimination validity we evaluated whether the scale and domains could discriminate those with different QOL: men with and without BPH, patients with BPH of different severity according to the IPSS (Total score has a range of 0 to 35: mild 0–7; moderate 8–19; and severe >20), and patients recruited from different settings inpatient, outpatient and community-based. T-test, correlation coefficient and profile analysis were then used to compare the short form of BPH-QLS with the original scale in terms of acceptability, reliability, and validity.

## Results

Of 163 86-item BPH-QLS questionnaires distributed, 79 (48.5%) were returned from inpatients; 45 (27.6%) from outpatients; and 39 (23.9%) from community-based patients (see additional file 2). The scale was re-administered to 40 randomly sampled outpatients one week after the first distribution. Thirty-one (31) of these patients returned their completed questionnaires. Of the 163 patients recruited, 125 completed the SF-36 whilst 153 completed the IPSS and all completed the new scale.

Additionally, a control group of 34 men with no past or current history of BPH randomly selected among the community were matched to 34 patients (cases) by age and educational level. The controls were also completed the questionnaire.

A total of 32 items were selected by using eight methods together. Among these methods, scoring by experts selected 36 items, coefficient of variation selected 46 items, discriminatory analysis selected 46 items, multiple regression selected 32 items, Cronbach'α coefficient selected 59 items, coefficient of correlation selected 53 items, factor analysis selected 54 items, and cluster analysis selected 46 items (see additional file 3). Finally, the short form of BPH-QLS consisted of 33 items (32 retained correlation items and 1 global QOL item) (table 1), including four new items: stop and start several times when you urinate? had a sensation of not emptying bladder completely after urinating, have to wait for urination to start and how would you score your quality of life including five domains (disease, physical, social, psychological, satisfaction)?

**Table 1:** Short-form of BPH-QLS (33-item scale)

new scale	Items
1	Had to urinate again less than 2 hours after you finished urinating
2	Strong urge to urinate
*3	Stop and start several times when you urinate?
4	Smaller or weaker force of your urinary stream
*5	Had a sensation of not emptying bladder completely after urinating
*6	Have to wait for urination to start
7	Dribbling and wetting pants a few minutes after finishing urinating
8	Getting up to urinate during the night
9	Have the symptoms of BPH brought trouble to your life?
10	Have you been worried that you would block up and not able to urinate?
11	How often have you worried about the urinary condition during the past 2 weeks?
12	Has the nocturia interfered with your sleep?
13	Has your sexual life been affected by the disease?
14	Do you feel uncomfortable when you going out or traveling, because of BPH?
15	If you have to spend the rest of your life with prostate symptoms just as they are now how would you feel about that?
16	Moving things heavier than 10 kg
17	Daily activities outside (e.g. shadowboxing)
18	To what extent can you take care of yourself
19	How about your sleep?
20	Have you given up some hobbies because of the illness?
21	Has your family life been interfered with by the illness?
22	Has your family responsibility been lost because of the illness?
23	Has the expectation from others fallen because of your illness?
24	Has your contact with friends reduced since your illness?
25	Have you been worried that therapy will cost so much money that you can't afford it?
26	Have you felt uneasy about your health?
27	Have you been worried about the outcome of the disease?
28	To what extent have you felt downhearted and depressed?
29	Do you look on yourself as a burden to the family and society?
30	Have you become more irritable than before?
31	Are you satisfied with your income?
32	Generally, are you satisfied with your health status?
*33	How would you score your quality of life including five domains(disease, physical, social, psychological, satisfaction)?(full mark is 100)__________

* New item

### Reliability

The test-retest CC was 0.858 for outpatients (not surgical), Cronbach'α coefficient was 0.952, providing evidence that the short form of BPH-QLS was stable and reliable in the light of generally recognized criteria and if good reliability is deemed as a test-retest CC of >0.7 and α >0.8[6].

### Validity

The content validity of the 33-item scale was validated with BPH-QLS, moreover, 18 relevant experts approved the final item pool.

Structure validity of the short-form of BPH-QLS gave a Kaiser-Meyer-Olkin test value of 0.858 and a significant Bartlette's test of sphericity (p < 0.001). A total of 12 common factors with the best interpretation were extracted after varimax rotation, and had a cumulative variance of 83.078% (see additional file 4). The 12 common factors were stratified into five domains, based on the conceptual model (Table 2).

**Table 2:** Construction of the short-form BPH-QLS

Domain (variance, %)	Common factor	Related Items	N items
Disease (47.501%)	1-symptoms of incontinence and effects of LUTS on daily life	1,2,4,5,8–15	15
	6-urine stoppage	3, 6	2
	8-dribbling after voiding	7	1
Physical (8.316%)	5-body activity	17,18	2
	10-sleep	19	1
	11-physical activity	16	1
Social (9.363%)	3-social function	22,23,24	3
	4-social activity	20,21	2
Psychological (14.899%)	2-emotional effects of disease	26–29	4
	9-worry about the disease	25	1
	12-temper	30	1
Satisfaction(3.001%)	7-life satisfaction	31,32	2

The structure validity was further assessed using correlation analysis of scores between domains, domains and the scale. The CCs for disease, physical, social, psychological and satisfaction with the scale were 0.828, 0.723, 0.830, 0.877, and 0.786, respectively. The CCs between domains were 0.424 for disease and physical; 0.515 for physical and social; 0.777 for social and psychological; and 0.612 for psychological and satisfaction.

Criterion validity of the short-form of BPH-QLS is summarized in table 3. The domains of the short-form of BPH-QLS and SF-36 were significantly correlated (p < 0.01) with CCs of 0.267~0.773, most of which were > 0.300. The disease domain of the new scale and that of the IPSS were also significantly correlated (p < 0.01; CC = 0.901).

**Table 3:** The CCs of the short-form BPH-QLS with other instruments

Short-form BPHQLS	SF-36	IPSS	IPSSQOL	74-item BPHQLS
Disease domain	0.595**	0.901**	0.755**	0.980**
Physical domain	0.663**	0.287**	0.262**	0.905**
Social domain	0.769**	0.400**	0.381**	0.938**
Psychological	0.742**	0.510**	0.506**	0.956**
Satisfaction	0.610**	0.411**	0.475**	0.864**
Total	0.822**	0.694**	0.609**	0.971**

**P < 0.01

**Figure 1:**
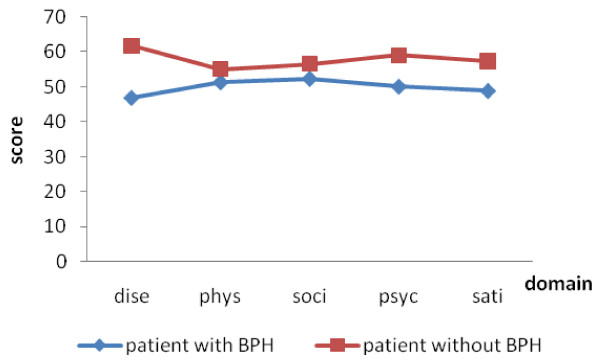
**The quality-of-life scores for patients with BPH or not**.

**Figure 2:**
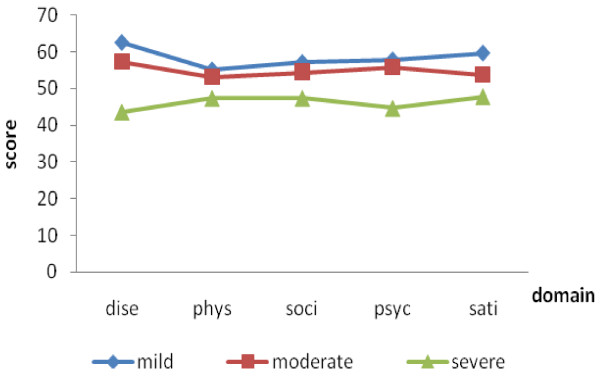
**The quality-of-life scores for different severity groups**.

**Figure 3:**
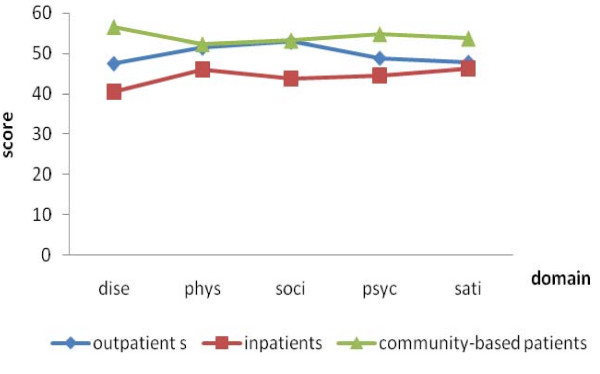
**The quality-of-life score for different sources**.

For discriminatory validity of the new scale, paired t-tests showed that scores for patients without BPH were significantly higher than men with BPH for all domains (Figure. 1). In terms of severity categorized by the IPSS (mild, moderate, and severe), ANOVA tests showed that patients with mild BPH had the highest score within the disease domain (p < 0.05). For the physical domain, mild and moderate groups showed higher scores than the severe group. However, in the social, psychological, satisfaction domain scores and the total score, the moderate group was not significant difference compared to the mild, and these two groups all exhibited higher score than that in the severe group (Figure. 2). We also compared the scores among community-based patients, outpatients and inpatients, and ANOVA showed a significant difference across the domains and scale scores of the three groups (p< 0.01). Compared between each, community-based patients had the highest scores in the disease, physical domains and the scale, while inpatients had the lowest. Outpatients and community-based patients had no significant difference in score for the physical and social domain, while inpatients had a lower score. For the satisfaction domain, outpatients and inpatients had similar scores but lower than community-based patients (Figure. 3).

### Comparison between the short scale and the full scale

The 74-item scale takes approximately 12–22 min to complete, while the short scale only 6–9 min. Out of 50 randomly recruited patients 49 (98%) considered the 33-item scale to be more acceptable than the 74-item scale.

Pearson's correlation analysis showed that there were significant correlations in each domain between short-form of BPH-QLS and BPH-QLS with CCs of 0.864–0.980 (table 3), and correlation of the total score between the two scales r^2 ^= 0.947. The short-form scale accounted for 94.7% of the explained variance of the 74-item one (table 4).

**Table 4:** The correlation between the 33-item and 74-item questionnaires

	Total original score ($\bar{X}$ ± *S*^)^	r	R^2^	*P*-value
74-item BPH-QLS	288.82 ± 45.66	0.971	0.947	0.000
33-item BPH-QLS	119.32 ± 22.61			

**Figure 4:**
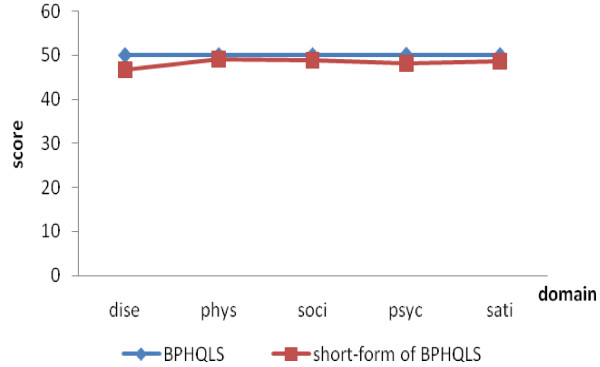
**Profiles of domains between the two scales**. dise-disease domain; physic-physical dimain; soci-social domain; psyc-psychological domain; sati-satisfaction domain

Profile analysis indicated that the two scales were not only parallel (F = 0.98, p = 0.419) and coincidence (F = 1.05, p = 0.307) but also of the same level (F = 2.00, p = 0.097) (Figure 4).

## Discussion

Quality of life measures have gained increasing attention as clinically relevant patient-centered endpoints in clinical trials. However, in a clinical setting, a lengthy quality of life scale is problematic for both the patient and the urologist. Short scale minimize a patient's time and effort, and thus increase a patient's willingness to complete the scale. The short-form BPH-QLS developed in our study was constructed based on WHO's definition of quality of life and a previously developed 74-item scale. In this study, we added some new items which were considered important by urologists and patients, while ignored in the 74-item scale, after consulting relevant published papers, experienced urologists and some patients. Taking the patient's educational level into account, some items were expressed in spoken language. For example, 'no emptying sensation after urination' change to 'had a sensation of not emptying bladder completely after urinating'.

Item reduction is a key technique in constructing the short scale. For different reduction methods get different results, item reduction was carried out from different angles as much as possible. The easier practice is to use systematic analysis and clinical experience to select items. This approach, is often influenced by the valuator's understanding of quality of life. Since there was the need to use objective methods to get more credible results[13], we used scoring by experts, coefficient of variation, discriminatory analysis, multiple regression, Cronbach'α coefficient, coefficient of correlation, factor analysis, and cluster analysis to objectively select items that were representative enough to assess the quality of life of BPH patients[2,9,12,13]. Coefficient of correlation, factor analysis and cluster analysis methods were used based on the relevant of the data structure from independence and representative aspect. According to the variation of the data structure, we used coefficient of variation, discriminate analysis, and stepwise regression analysis to select items from sensitivity and the importance aspect [12]. Cronbach'α coefficient was used to item reduction on the basis of the item internal consistency[13]. The previous study of BPHQLS did not use scoring by experts and Cronbach'α coefficient to select items. The use of experts is considered an important method for the reduction of items. In our study, we used 12 experts with over 10 years of experience. This was consistent with suggestions by Sun et al [14]. Furthermore, the use of Cronbach'α coefficient was important in the selection of items with higher internal consistency and stability, thereby resulting in the selection of items that enhanced the assessment quality of life.

The clinical experience was used to identify the items that did not meet the statistic criteria but considered important from a clinical perspective [2]. For example, question "has your sexual life been affected by the disease?" which was selected by only 5 of the 8 methods was considered to retain since all the foreign BPH-QOL scales included sexual life items and some even contained more than one third, while response rate of this item was very low because of the Chinese tradition to assign much importance on the family. Then, all the items were sensitive, independent and representative, according to the construction strategy and item selection methods.

The compliance of subjects is a very important indicator of the effectiveness of a scale, and this is often influenced by the length of the scale (the shorter the scale the easier it is to administer). Currently, IPSS, BII and BPH-HRQOL9 are widely used in clinical setting because they are short, and are reported to have completion rates of over 85%[15,16]. In our study, the acceptance rate for the new short scale was 98%, and the time which the short scale takes was much less than the original long scale. Expressing some of the items in a spoken language format also enhanced acceptability and a higher rate of completion.

In our study, not only were the reliability and validity of the scale tested, the comparison was also used to assess the differences between the short-form of BPH-QLS and the original long scale. The results indicated that the new scale were as good as the original scale. Although there were fewer items in the new scale, the content validity seemed better than the original one by the experts' subjective evaluation and the additional self-assessment globe QOL item. The new short scale accounted for 94.7% of the explained variance of the 74-item scale after half of the items were reduced. The CCs of the short-form of BPH-QLS with SF-36, IPSS, IPSSQOL score and 74-item BPH-QLS were all high (0.822, 0.901, 0.775, and 0.971, respectively), showing good criterion validity. Similar to the 74-item BPH-QLS, the 33-item scale could also discriminate the following kinds of persons: patients with BPH and those without BPH; patients with different degree of symptoms; in-patients, out-patients, and community patients [17].

## Conclusion

This study used scientifically sounding strategy to construct a short-form of BPH-QLS for Chinese men. The new scale reliable and valid with improved acceptance. Hence, we concluded that the reliability and validity of the short form of BPH-QLS is close to those of the 74-item BPH-QLS. It should be a good choice in clinical practice for its greater compliance and clinical feasibility.

## Abbreviations

BPH-QLS: A Quality of Life Scale for patients with BPH Prior Test Version; QOL: quality of life; DAN-PSS-1: Danish Prostate Symptom Score; ICSmaleSF: International Continence Society male questionnaire short-form; SF-36: The Medical Outcomes Study 36-Item Short-Form Health Survey; BPH-QoL: A Quality of Life Scale for patients with BPH; WHOQoL-100: the WHO Quality of Life.

## Competing interests

The authors declare that they have no competing interests.

## Authors' contributions

YG collected data and drafted the whole manuscript. JS was involved in conception and designed the study. MH contributed in interpretation of data and in selection of patients, ZS design the study and he was the soul of this article. All authors read and approved the final manuscript.

## Supplementary Material

Additional file 1 **86-item scale (74-item BPH-QLS +12 new items).** The file provided represent the initial draft-item pool (86 items) which included 12 new items and 74 items of BPH-QLS. Click here for file

Additional file 2 **The demographic structures of the sample. **The table provided represents the baseline characteristics for patients who were in the three different sources. Click here for file

Additional file 3 **The process of the item selection.** The table showed the results in selecting items by eight statistical methods of analysis. Click here for file

Additional file 4 **Factor loadings of the short form of BPH-QLS. **The table showed the factor analysis of the short form of BPH-QLS. Click here for file
