# Supplementary material for: Construction and validation of a short-form Quality-Of-Life Scale for Chinese Patients with Benign Prostatic Hyperplasia
Source: Health Qual Life Outcomes. 2009 Mar 17;7:24. doi: 10.1186/1477-7525-7-24 (PMC2678090; doi:10.1186/1477-7525-7-24)
Supplement: Additional file 1 — 86-item scale (74-item BPH-QLS +12 new items). The file provided represent the initial draft-item pool (86 items) which included 12 new items and 74 items of BPH-QLS. [file 1477-7525-7-24-S1.doc]

**Additional file 1**

**86-item scale (74-item BPH-QLS +12 new items):**

*1a.Had a sensation of not emptying bladder completely after urinating

*1b. Bother due to no sensation to empty bladder after urinating

2a. Had to urinate again less than 2 hours after you finished urinating

2b.Bother due to frequent urination

*3a.Stop and start several times when you urinate?

*3b.Bother due to stopping and starting several times when urinating

4a.Strong urge to urinate

4b.Bother due to the urgency

5a.Smaller or weaker force of your urinary stream

5b.Bother due to smaller or weaker force of urinary stream

6a.Strain to continue urinating and duration of urination is too long

6b.Bother due to straining to continue urinating and duration of urination is too long

7a.Getting up to urinate during the night

7b.Bother due to getting up to urinate during the night

*8a.Have to wait for urination to start

*8b.Bother due to waiting for urination to start

9a.Painful or burning sensation during urination

9b.Bother due to painful or burning sensation during urination

10a.Leakage of urine (e.g. When you are coughing)

10b.Bother due to leakage of urine

11a.Dribbling and wetting pants a few minutes after finishing urinating

11b.Bother due to dribbling and wetting pants a few minutes after finishing urinating

*12a.Leak urine when you asleep

*12b.Bother due to urine leakage when you asleep

25. Have the symptoms of BPH brought trouble to your life?

26. Have you been worried that you would block up and not be able to urinate?

27. How often have you worried about the urinary condition during the past 2 weeks?

28. Has the nocturia interfered with your sleep?

29. Do you consider the pattern of urination as a bother to your daily life?

30. Has your sexual life been affected by the disease?

31. Do you feel uncomfortable when you going out or traveling, because of BPH?

32. How worried or concerned have you been about having or getting prostate cancer?

33. If you have to spend the rest of your life with prostate symptoms, just as they are now how would you feel about that?

34. Have you been worried that therapy will cost so much money that you can’t afford it?

35. Are you satisfied with your sexual life?

**B. The following items are about activities you might do during a typical day. Does your health now limit you in these activities** in the **last two weeks? If so, how much?**

36. Moving things heavier than 10 kg

37. Carrying things of about 2.5–5 kg

38. Buying articles for daily use

39. Daily activities outside (e.g. shadowboxing)

40. Walk by yourself

41. Go upstairs or downstairs/climb slope or go downhill by yourself

42. Daily physical activity (stoop, bend your knees or squat on heels)

43. To what extent can you take care of yourself?

44. How much of the time do you feel fresh and rested?

45. How much of the time have you had enough energy to do anything that you wanted to do?

46. How about your sleep?

47. Are you satisfied with your sleep?

48. How about your appetite?

49. Are you satisfied with your appetite?

50. Do you have pain?

51. Does the pain interfere with your daily life or work?

*52. Have you had problems with your ability to concentrate over the past week to two?

*53. How well do you remember things that happened several years ago?

*54. How well do you remember things that happened very recently?

**C. During the past 2 weeks, have you had any of the following problems with your work or other regular daily activities as a result of your disease?**

55. To what extent has your working ability been affected by the illness?

56. Have you had any difficulties with your work or leisure (e.g. reading newspapers?)

57. To what extent has your working time been reduced by BPH?

58. Has your work been interfered with by the disease, e.g. finishing a particular job?

59. Has your leisure been interfered with by the disease?

60. Have you had any difficulty when doing housework?

61. Have you given up some hobbies because of the illness?

62. Has your family life been interfered with by the illness?

63. Has your family responsibility been lost because of the illness?

64. Has the expectation from others fallen because of your illness?

65. Has your contact with friends reduced since your illness?

66. To what extent have your friends become estranged from you because of your illness?

67. Have you tried to avoid attending meetings or other situations with many people?

**D. During the past 2 weeks, have you had any of the following affectional problems as a result of your disease?**

68. Have you felt uneasy about your health?

69. Have you been worried about the outcome of the disease?

70. Are you afraid of becoming a useless man who needs the care of others?

71. To what extent have your felt downhearted and depressed?

72. Do you look on yourself as a burden to the family and society?

73. Have you thought that you are not as good as others?

74. Are you disappointed with your health?

75. Have you become more irritable than before?

76. Do you often quarrel with others?

77. How much of the time have you found nothing interesting in life?

**E Satisfaction**

78.Are you satisfied with your family condition?

79. Are you satisfied with your working condition?

80. Are you satisfied with your daily living condition?

81. Are you satisfied with your physical status?

83. Are you satisfied with your income?

82. Are you satisfied with your psychological status?

84. Are you satisfied with your relationship with other people?

85. Generally, are you satisfied with your health status?

*86. How would you score your quality of life using five domains (disease, physical, social, psychological, satisfaction)? (Full mark is 100)

* New item
