# Supplementary material for: Construction and validation of a short-form Quality-Of-Life Scale for Chinese Patients with Benign Prostatic Hyperplasia
Source: Health Qual Life Outcomes. 2009 Mar 17;7:24. doi: 10.1186/1477-7525-7-24 (PMC2678090; doi:10.1186/1477-7525-7-24)
Supplement: Additional file 2 — The demographic structures of the sample. The table provided represents the baseline characteristics for patients who were in the three different sources. [file 1477-7525-7-24-S2.doc]

**Additional file 2**

**The demographic structures of the sample**

|  | | Outpatient | |  | Inpatient | | |  | Community | |  | Total | | |
| --- | --- | --- | --- | --- | --- | --- | --- | --- | --- | --- | --- | --- | --- | --- |
|  |  | N | （%） |  | N | （%） | |  | N | （%） |  | N | （%） | |
| Age | 50～ | 8 | 17.8 |  | 16 | | 20.3 |  | 3 | 7.7 |  | 27 | | 16.6 |
|  | 60～ | 23 | 51.1 |  | 23 | | 29.1 |  | 23 | 59.0 |  | 69 | | 42.3 |
|  | 70～ | 13 | 28.9 |  | 32 | | 40.5 |  | 11 | 28.2 |  | 56 | | 34.4 |
|  | 80～ | 1 | 2.2 |  | 8 | | 10.1 |  | 2 | 5.1 |  | 11 | | 6.7 |
|  | Total | 45 | 100.0 |  | 79 | | 100.0 |  | 39 | 100.0 |  | 163 | | 100.0 |
| Marital status | First marriage | 40 | 90.9 |  | 65 | | 82.3 |  | 36 | 92.3 |  | 141 | | 87.0 |
|  | Remarried | 1 | 2.3 |  | 5 | | 6.3 |  | 1 | 2.6 |  | 7 | | 4.3 |
|  | Divorce | 2 | 4.5 |  | 0 | | 0.0 |  | 0 | 0.0 |  | 2 | | 1.2 |
|  | Lose spouse | 1 | 2.3 |  | 9 | | 11.4 |  | 2 | 5.1 |  | 12 | | 7.4 |
|  | Total | 44* | 100.0 |  | 79 | | 100.0 |  | 39 | 100.0 |  | 162 | | 100.0 |
| Education level | Illiteracy/  semiliterate | 2 | 4.5 |  | 7 | | 9.1 |  | 1 | 2.6 |  | 10 | | 6.3 |
|  | Primary school | 10 | 22.7 |  | 17 | | 22.1 |  | 4 | 10.5 |  | 31 | | 19.5 |
|  | Junior high school | 8 | 18.2 |  | 16 | | 20.8 |  | 8 | 21.1 |  | 32 | | 20.1 |
|  | High school | 8 | 18.2 |  | 17 | | 22.1 |  | 12 | 31.6 |  | 37 | | 23.3 |
|  | University and above | 16 | 36.4 |  | 20 | | 26.0 |  | 13 | 34.2 |  | 49 | | 30.8 |
|  | Total | 44* | 100.0 |  | 77* | | 100.0 |  | 38* | 100.0 |  | 159 | | 100.0 |
| Habitation | Living alone | 1 | 2.3 |  | 9 | | 11.4 |  | 1 | 2.6 |  | 11 | | 6.8 |
|  | With spouse | 32 | 72.7 |  | 43 | | 54.4 |  | 31 | 79.5 |  | 106 | | 65.4 |
|  | With child | 10 | 22.7 |  | 9 | | 11.4 |  | 1 | 2.6 |  | 20 | | 12.3 |
|  | With families | 1 | 2.3 |  | 18 | | 22.8 |  | 6 | 15.4 |  | 25 | | 15.4 |
|  | Total | 44* | 100.0 |  | 79 | | 100.0 |  | 39 | 100.0 |  | 162 | | 100.0 |

* having missing value
