# Supplementary material for: Construction and validation of a short-form Quality-Of-Life Scale for Chinese Patients with Benign Prostatic Hyperplasia
Source: Health Qual Life Outcomes. 2009 Mar 17;7:24. doi: 10.1186/1477-7525-7-24 (PMC2678090; doi:10.1186/1477-7525-7-24)
Supplement: Additional file 3 — The process of the item selection. The table showed the results in selecting items by eight statistical methods of analysis. [file 1477-7525-7-24-S3.doc]

**Additional file 3**

The process of the item selection

| Item | Experts scoring | Variation coefficient | Discriminatory analysis | Multiple regression | Cronbach’α coefficient | Correlation coefficient | Factor analysis | Cluster analysis | Keep |
| --- | --- | --- | --- | --- | --- | --- | --- | --- | --- |
| *1a | √ | √ | √ |  | √ | √ | √ | √ | 7 |
| *1b |  | √ | √ |  | √ |  | √ |  | 4 |
| 2a | √ | √ | √ | √ | √ | √ | √ | √ | 8 |
| 2b |  | √ | √ |  | √ |  | √ |  | 4 |
| *3a | √ | √ | √ | √ | √ | √ | √ | √ | 8 |
| *3b |  | √ | √ |  | √ |  | √ |  | 4 |
| 4a | √ | √ | √ | √ | √ | √ |  | √ | 8 |
| 4b |  | √ | √ |  | √ |  | √ |  | 4 |
| 5a | √ | √ | √ |  | √ | √ | √ | √ | 7 |
| 5b |  | √ | √ | √ | √ |  | √ |  | 5 |
| 6a | √ | √ | √ |  |  |  | √ |  | 4 |
| 6b |  | √ | √ |  | √ |  | √ |  | 4 |
| 7a | √ | √ | √ |  | √ | √ | √ | √ | 7 |
| 7b |  | √ | √ |  | √ |  | √ | √ | 5 |
| *8a | √ | √ | √ |  |  | √ | √ | √ | 6 |
| *8b |  | √ | √ | √ | √ |  | √ |  | 5 |
| 9a |  | √ | √ | √ |  | √ | √ |  | 5 |
| 9b |  | √ | √ | √ | √ |  |  |  | 4 |
| 10a | √ |  |  |  |  | √ | √ | √ | 4 |
| 10b |  |  |  |  |  |  | √ |  | 1 |
| 11a | √ | √ | √ | √ |  | √ | √ | √ | 7 |
| 11b |  | √ | √ |  | √ |  | √ |  | 4 |
| *12a |  |  |  | √ |  | √ | √ |  | 3 |
| *12b |  |  |  | √ |  |  | √ |  | 2 |
| 25 | √ | √ | √ |  | √ | √ | √ | √ | 7 |
| 26 | √ | √ | √ | √ | √ | √ |  | √ | 7 |
| 27 |  | √ | √ |  | √ | √ |  | √ | 5 |
| 28 | √ | √ | √ |  | √ | √ |  | √ | 6 |
| 29 |  | √ | √ |  | √ |  | √ |  | 4 |
| 30 | √ |  |  |  | √ | √ | √ | √ | 5 |
| 31 |  | √ | √ |  | √ | √ | √ | √ | 6 |
| 32 | √ |  |  |  |  | √ |  | √ | 3 |
| 33 |  | √ | √ |  |  | √ | √ | √ | 5 |
| 34 |  | √ | √ | √ |  | √ | √ | √ | 6 |
| 35 |  |  |  |  |  |  |  | √ | 1 |
| 36 |  | √ | √ |  | √ | √ | √ | √ | 6 |
| 37 | √ |  |  |  | √ | √ | √ |  | 4 |
| 38 | √ |  |  | √ | √ |  | √ |  | 4 |
| 39 | √ |  |  | √ | √ | √ | √ |  | 5 |
| 40 |  |  |  |  | √ |  | √ |  | 3 |
| 41 | √ |  |  | √ | √ |  | √ |  | 4 |
| 42 | √ |  |  |  | √ |  |  |  | 2 |
| 43 | √ |  |  |  | √ | √ | √ | √ | 5 |
| 44 | √ |  |  |  | √ | √ |  | √ | 4 |
| 45 |  |  |  | √ | √ | √ |  |  | 3 |
| 46 | √ | √ | √ |  | √ | √ |  | √ | 6 |
| 47 |  | √ | √ | √ | √ |  |  | √ | 5 |
| 48 |  |  |  |  |  | √ | √ |  | 2 |
| 49 |  |  |  |  |  |  | √ |  | 1 |
| 50 |  | √ | √ |  | √ | √ | √ |  | 5 |
| 51 |  | √ | √ |  | √ |  | √ |  | 4 |
| *52 |  |  |  |  | √ | √ |  |  | 2 |
| *53 |  |  |  |  |  | √ | √ |  | 2 |
| *54 |  |  |  | √ |  | √ | √ | √ | 4 |
| 55 |  |  |  |  |  |  |  | √ | 1 |
| 56 | √ |  |  | √ | √ | √ |  |  | 4 |
| 57 |  |  |  |  |  |  |  | √ | 1 |
| 58 |  |  |  |  |  |  |  | √ | 1 |
| 59 |  | √ | √ |  | √ |  |  |  | 3 |
| 60 | √ |  |  |  | √ | √ |  | √ | 4 |
| 61 | √ |  | √ | √ | √ | √ |  | √ | 6 |
| 62 | √ | √ | √ |  | √ | √ |  | √ | 6 |
| 63 |  | √ | √ |  | √ | √ | √ |  | 5 |
| 64 |  | √ | √ |  | √ | √ | √ |  | 5 |
| 65 | √ |  |  | √ | √ | √ |  | √ | 5 |
| 66 |  |  |  |  | √ |  | √ |  | 2 |
| 67 | √ | √ | √ |  |  | √ |  | √ | 5 |
| 68 | √ | √ | √ | √ | √ | √ | √ | √ | 8 |
| 69 | √ | √ | √ |  | √ | √ | √ | √ | 7 |
| 70 |  | √ | √ |  | √ |  | √ |  | 4 |
| 71 | √ | √ | √ | √ | √ |  | √ | √ | 7 |
| 72 | √ | √ | √ | √ | √ | √ | √ | √ | 8 |
| 73 |  |  |  | √ | √ |  | √ |  | 3 |
| 74 |  |  | √ | √ | √ | √ |  | √ | 5 |
| 75 | √ |  | √ |  | √ | √ | √ | √ | 6 |
| 76 |  |  |  |  |  | √ | √ |  | 2 |
| 77 | √ |  |  | √ |  | √ |  | √ | 4 |
| 78 |  |  |  |  |  | √ | √ |  | 2 |
| 79 |  |  |  |  |  |  |  | √ | 1 |
| 80 |  |  |  | √ | √ | √ | √ | √ | 5 |
| 81 |  | √ |  |  | √ |  |  |  | 2 |
| 82 |  |  |  | √ | √ | √ |  | √ | 4 |
| 83 |  | √ |  | √ | √ | √ | √ | √ | 6 |
| 84 |  |  |  |  |  | √ |  |  | 1 |
| 85 | √ | √ |  | √ | √ | √ |  | √ | 6 |
| *86 |  |  |  |  |  |  |  |  |  |

* new item
