# Supplementary material for: Construction and validation of a short-form Quality-Of-Life Scale for Chinese Patients with Benign Prostatic Hyperplasia
Source: Health Qual Life Outcomes. 2009 Mar 17;7:24. doi: 10.1186/1477-7525-7-24 (PMC2678090; doi:10.1186/1477-7525-7-24)
Supplement: Additional file 4 — Factor loadings of the short form of BPH-QLS. The table showed the factor analysis of the short form of BPH-QLS. [file 1477-7525-7-24-S4.doc]

**Additional file 4**

**Factor loadings of the short-**form of BPH-QLS

| Disease domain (47.501％) | Factor 1 Lowe urinary tract symptoms and the impact | 8. Getting up to urinate during the night | .846 |
| --- | --- | --- | --- |
|  |  | 1. Had to urinate again less than 2 hours after you finished urinating | .801 |
|  |  | 12. Has the nocturia interfered with your sleep? | .781 |
|  |  | 14. Do you feel uncomfortable when you going out or traveling, because of BPH? | .760 |
|  |  | 9. Have the symptoms of BPH brought trouble to your life? | .725 |
|  |  | 15. If you have to spend the rest of your life with prostate symptoms just as they are now how would you feel about that? | .679 |
|  |  | 2. Strong urge to urinate | .656 |
|  |  | 11. How often have you worried about the urinary condition during the past 2 weeks? | .626 |
|  |  | 4. Smaller or weaker force of your urinary stream | .589 |
|  |  | 10. Have you been worried that you would block up and not able to urinate? | .569 |
|  |  | 5. Had a sensation of not emptying bladder completely after urinating | .560 |
|  |  | 13. Has your sexual life been affected by the disease? | .557 |
|  | Factor 6  Urinate stopping | 6. Have to wait for urination to start | .719 |
|  |  | 3. Stop and start several times when you urinate? | .698 |
|  | Factor8 Dribbling after finishing urinating | 7. Dribbling and wetting pants a few minutes after finishing urinating | .804 |
| Physical domain  （8.316％） | Factor 5  Body activity | 17. Daily activities outside (e.g. shadowboxing) | .885 |
|  |  | 18. To what extent can you take care of yourself | .652 |
|  | Factor 10 Sleep | 19. How about your sleep? | .834 |
|  | Factor 11  Physical activity | 16. Moving things heavier than 10 kg | .869 |
| Social domain（9.363％） | Factor 3  Society role | 23. Has the expectation from others fallen because of your illness? | .830 |
|  |  | 24. Has your contact with friends reduced since your illness? | .655 |
|  |  | 22. Has your family responsibility been lost because of the illness? | .626 |
|  | Factor4 Disease impact the social activity | 20. Have you given up some hobbies because of the illness? | .776 |
|  |  | 21. Has your family life been interfered with by the illness? | .676 |
| Psychological  domain（14.899%） | Factor 2 Emotion | 26. Have you felt uneasy about your health? | .756 |
|  |  | 27. Have you been worried about the outcome of the disease? | .731 |
|  |  | 28. To what extent have you felt downhearted and depressed? | .518 |
|  |  | 29. Do you look on yourself as a burden to the family and society? | .506 |
|  | Factor 12 Temper | 30. Have you become more irritable than before? | .657 |
|  | Factor 9 Worry about the disease | 25. Have you been worried that therapy will cost so much money that you can’t afford it? | .649 |
| Satisfaction  domain  （3.001％） | Factor 7 Satisfaction | 31. Are you satisfied with your income? | .823 |
|  |  | 32. Generally, are you satisfied with your health status? | .529 |
